# Deep Convolutional Generative Adversarial Network with LSTM for ECG Denoising

**DOI:** 10.1155/2023/6737102

**Published:** 2023-02-10

**Authors:** Huidong Wang, Yurun Ma, Aihua Zhang, Dongmei Lin, Yusheng Qi, Jiaqi Li

**Affiliations:** ^1^College of Electrical and Information Engineering, Lanzhou University of Technology, Lanzhou 730050, China; ^2^Key Laboratory of Gansu Advanced Control for Industrial Processes, Lanzhou University of Technology, Lanzhou 730050, China; ^3^National Demonstration Center for Experimental Electrical and Control Engineering Education, Lanzhou University of Technology, Lanzhou 730050, China

## Abstract

The electrocardiogram (ECG), as an essential basis for the diagnosis of cardiovascular diseases, is usually disturbed by various noise. To obtain accurate human physiological information from ECG, the denoising and reconstruction of ECG are critical. In this paper, we proposed an ECG denoising method referred to as LSTM-DCGAN which is based on an improved generative adversarial network (GAN). The overall network structure is composed of multiple layers of convolutional networks. Furthermore, the convolutional features can be connected to their time series order dependence by adding LSTM layers after each convolutional layer. To verify the effectiveness and the denoising performance of the improved network structure, we test the proposed algorithm on the famous MIT-BIH Arrhythmia Database with different levels of noise from the MIT-BIH Noise Stress Test Database. Experimental results show that our method can remove the single noise and the mixed noise while retaining the complete ECG information. For the mixed noise removal, the average SNR_imp_, RMSE, and PRD are 19.254 dB, 0.028, and 10.350, respectively. Compared with the state-of-the-art methods, DCGAN, and the LSTM-GAN methods, our method obtains the higher SNR_imp_ and the lower RMSE and PRD scores. These results suggest that the proposed LSTM-DCGAN approach has a significant advantage for ECG processing and application in complex scenes.

## 1. Introduction

Cardiovascular diseases pose a serious threat to human health, especially due to the sudden and intermittent nature of heart disease; therefore, routine monitoring is the best strategy to diagnose these diseases. As a “gold standard” for assessing heart conditions, ECG has been widely used in clinical diagnosis and daily personal monitoring. However, the captured ECG signals are usually interfered by the noise from the equipment, environment, and human movement, which may lead to a failed diagnosis. Therefore, it is crucial to remove the interference from the recorded ECG and reconstruct a clean ECG.

Generally speaking, the noise affecting the ECG signal mainly includes the electrode motion (EM), the muscle artifact (MA), and the baseline wander (BW). Many scholars have conducted in-depth research on removing noise from ECG signals and proposed some efficient methods. For example, filtering [1–5], wavelet [6], and empirical mode decomposition (EMD) [7] are typical ECG denoising methods. Filtering methods include finite impulse response (FIR) filter [1], infinite impulse response (IIR) filter [2], adaptive filter [3], Kalman filter [4], and Wiener filter [5]. Among them, the FIR and IIR filters are not applicable when the frequency domains of the ECG and noise signals overlap. The adaptive filter requires the reference signal, and the Wiener filter is difficult to remove the complex noise. For the wavelet methods, the choices of wavelet function and threshold function have a great influence on the denoising performance. To reduce the end point effect and modal aliasing in the traditional EMD algorithms, the ensemble empirical mode decomposition (EEMD) [8] and variational mode decomposition (VMD) [9] methods are published. However, it is necessary to artificially add Gaussian white noise in order to use the EEMD approach, and the parameters of the VMD technique are sensitive to varied inputs. There are also several EMD paired with wavelet algorithms for ECG denoising algorithms, but most of them are only useful for certain types of noise reduction [10–12]. In recent years, some researchers have employed machine learning techniques for ECG denoising. Their works can be roughly classified into two categories: (1) improving existing algorithms by machine learning methods for parameter optimization [13, 14] and (2) building the ECG denoising model to decompose and reconstruct the ECG using a neural network [15–19]. To reduce baseline drift, Sun et al. proposed combining error backpropagation neural network and VMD technique [13]. Wang et al. proposed an ECG signal denoising algorithm based on a supervised deep factor analysis model [14]. Xiong et al. used the denoise autoencoder (DAE) for the denoising and compression of ECG signals [15]. Chiang et al. completed the denoising and compression of ECG based on the fully convolutional network denoising autoencoder (FCN-DAE). The fully convolutional network is applied to preserve the detailed information of ECG as much as possible [16]. Dasan and Panneerselvam accomplished ECG compression and reconstruction by adding a long short-term memory (LSTM) network to the encoder tail of a convolutional denoising autoencoder [17]. Wang et al. created the ECG denoising algorithm based on GAN, in which the new loss function and deep neural networks (DNN) were used [18]. However, using DNN is more likely to cause QRS wave group distortion [16]. Therefore, some researchers achieved the denoising model using the residual network structure in GAN [19]. Additionally, the GAN with a CNN-based discriminator and an LSTM-based generator is used to generate ECG [20]. Although the convolutional neural network (CNN) does not cut the waveform amplitude, it reconstructs the denoised signal from the convolutional features; each convolution is independent and lacks mutual information. Because of the significant individual differences, the large pathological differences, and the complex behavioral environments, it is still difficult to remove as much noise as possible while keeping the ECG information intact. Up to now, researchers are still working on developing the ECG denoising approaches which can be integrated into the actual monitoring equipment.

In consideration of both the global information and the local time domain characteristics of ECG, we design the LSTM-DCGAN for ECG denoising. For the local feature capturing, the CNN with a smaller receptive field is used. For the global information reserving, we adopt the GAN structure to ensure that the denoised ECG signal achieves the same data distribution as the clean ECG signal. At the same time, the GAN is improved by adding the LSTM layer after each convolutional layer, which can correlate its sequential dependencies on time series and strengthen its global information capability. The following text will introduce the proposed ECG denoising network from the aspects of methods, experiments, results, and discussion in detail.

## 2. Materials and Methods

### 2.1. Denoising Model Based on GAN

The GAN is a structure that estimates a generative model through an adversarial process and is composed of a generator (*G*) and a discriminator (*D*) [21]. GAN trains two models: the generator generates the observed data *G*(*z*) by assigning some noise variable *z*, and the discriminator distinguishes between the true data *x* and the generated data *G*(*z*). The optimization goal of the generator is to maximize *D*(*G*(*z*)) such that the discriminator incorrectly discriminates the generated data. The optimization goal of the discriminator is to maximize *D*(*x*) as well as minimize *D*(*G*(*z*)) to improve its classification performance. Generator and discriminator both improve their performance through adversarial training. This adversarial process is described as a min-max game with the value function *V*(*D*, *G*) shown in Equation (1), until the generator and discriminator have sufficient capacity and the optimization of both reaches a critical state so the generator reproduces the real data distribution.

(1) $$\begin{array}{c} \underset{G}{\min}\underset{D}{\max}V\left(D,G\right)=E_{x∼p_{\text{data}}\left(x\right)}\left[\log D\left(x\right)\right]+E_{z∼p_{z}\left(z\right)}\left[\log\left(1-D\left(G\left(z\right)\right)\right)\right], \end{array}$$

where *P*_data_(*x*) represents the distribution of real data samples *x*, and *P*_*z*_(*z*) represents the distribution of the input variable *z* of the generator, and *𝔼* represents the mathematical expectation about the distribution specified in the subscript.

The noisy ECG signal can be described by

(2) $$\begin{array}{c} \tilde{x}=x+n, \end{array}$$

where $\tilde{x}$ is the ECG signal with noise, *x* is the clean ECG signal, and *n* is noise.

**Figure 1:**
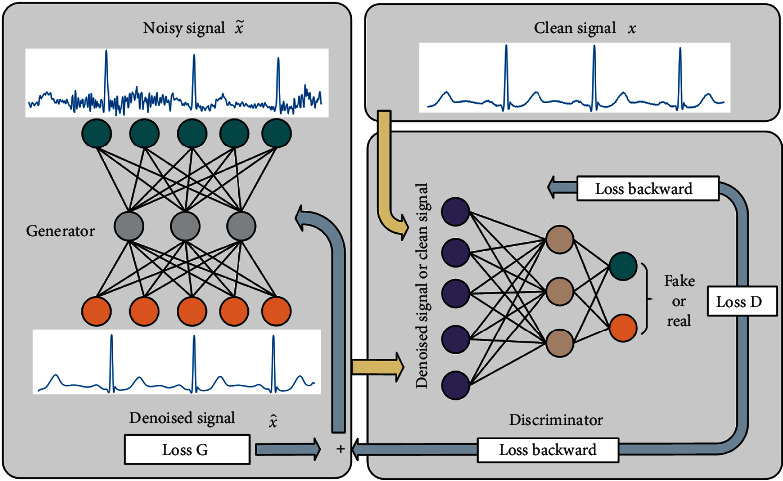
Denoising model based on GAN.

As shown in Figure 1, in the adversarial denoising model, the generator takes the noisy ECG signal $\tilde{x}$ as input and outputs the denoised ECG signal $G\left(\tilde{x}\right)$. The input of the discriminator contains the denoised ECG signal $G\left(\tilde{x}\right)$ or the original clean ECG signal *x*, and the discriminator outputs probabilistically by judging whether the input originates from the denoised signal $G\left(\tilde{x}\right)$ or the clean signal *x*. The ECG denoising problem can be described as obtaining *x* from the noisy ECG signal $\tilde{x}$.

(3) $$\begin{array}{c} G\left(\tilde{x}\right)=\hat{x}\underset{\text{a}.\text{c}.\text{a}.\text{p}}{⟶}x=\tilde{x}-n, \end{array}$$

where a.c.a.p is short for “as close as possible” and $\hat{x}$ is the denoised signal.

The discriminator continuously improves its ability by passing the loss *D* backward to minimize the discriminator *D*(*G*(*x*˜)). The optimization goal of the generator is to maximize the discriminator $D\left(G\left(\tilde{x}\right)\right)$ by updating the network parameters using loss *D* and loss *G* together, so that the generated denoised signal $\hat{x}$ is as close as possible to the original signal *x*. Both the generator and the discriminator are trained through a continuous game, until the optimization of both the generator and the discriminator reaches a critical state. The denoised signal $\hat{x}$ is almost the same as the clean signal *x*, so the generator has denoising capability.

Because the denoising performance of GAN is determined by the structures of the generator and discriminator, we propose the improved GAN for ECG denoising whose generator structure and discriminator structure are, respectively, described in 2.2 and 2.3.

### 2.2. Generator and Value Function

**Figure 2:**
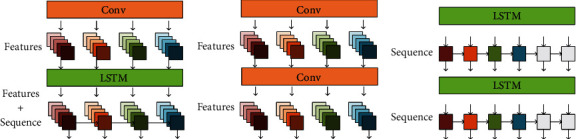
Three different structures of generators.

It is generally believed that MA and EM noise affect the detailed waveform of ECG. The BW noise affects the overall trend of the ECG signal, which is reflected in the drift of the baseline. Thus, we design the GAN by integrating the deep convolutional generative adversarial networks (DCGAN) [22] and the LSTM [23]. On one hand, we apply the DCGAN replacing the upsampling steps in the original GAN with the fractional-stride convolution to capture the local features and achieve feature recombination for the ECG details. On the other hand, the LSTM controlled by forget gates, input gates, and output gates is used to preserve the global time domain information of ECG. In this way, we design an iterative generator structure consisting of convolutional layers and LSTM layers, which is called CNN-LSTM structure and shown in Figure 2(a). To verify the effectiveness of the designed structure, in 3.1.1, we compare the denoising performance of the CNN-LSTM with the convolutional structure shown in Figure 2(b) and the LSTM structure shown in Figure 2(c).

**Figure 3:**
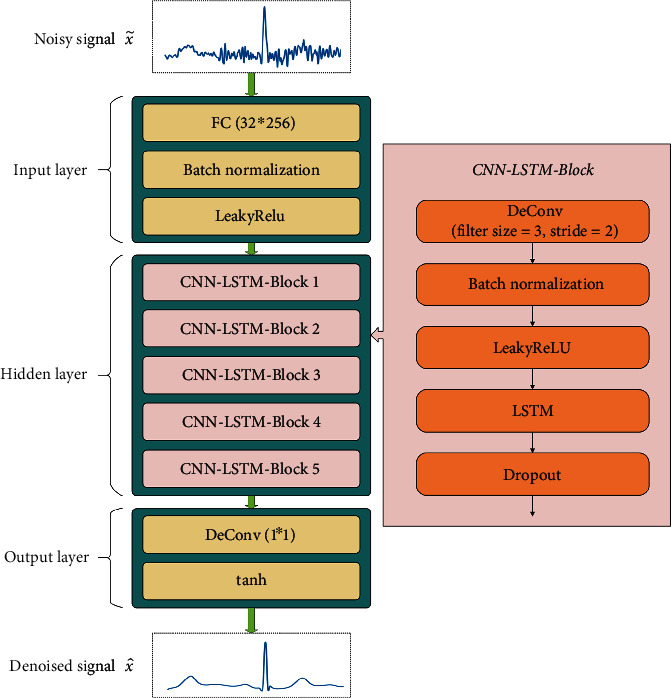
Generator structure.

As shown in Figure 3, the generator consists of an input layer, a hidden layer based on the CNN-LSTM-Block structure, and an output layer. The input layer consists of a fully connected (FC) layer of size 32^∗^256, a batch normalization layer, and a LeakyReLU activation layer. ReLU sets all negative values to zero, and the gradient of neurons is always zero. For the training of GAN, ReLU makes the training fragile and a large number of neurons die. Instead, LeakyReLU assigns a nonzero fixed slope *α* to all negative values, ensuring that the gradient can flow through the entire architecture. The LeakyReLU activation function is shown in Equation (4). The slope *α* of PReLU is a nonfixed learnable parameter. The hidden layer consists of 5 CNN-LSTM-Blocks with 128-64-40-20-40 nodes. Each CNN-LSTM-Block consists of one deconvolution layer (filter size = 16 and stride = 2) and one LSTM layer. Batch normalization and LeakyReLU layers are added after each deconvolution layer. After each LSTM layer, a dropout layer with a rate of 0.5 and tanh activation function is added. Tanh activation function is shown in Equation (5). In the output layer, a deconvolution layer of size 1^∗^1 is used to recover the data length of 1^∗^1024.

(4) LeakyReLUx=x,x≥0,αx,x<0,

(5) $$\begin{array}{c} \tanh\left(x\right)=\frac{e^{x}-e^{-x}}{e^{x}+e^{-x}}. \end{array}$$

The optimization objective of the generator is to minimize log(1 − *D*(*G*(*x*˜))), and the value function *V*(*G*) is shown in Equation (6). In addition, we focus on the difference between the global and the local of the model by adding distance function (*L*_dist_) and the maximum local difference (*L*_max_), where *L*_dist_ and *L*_max_ are defined in Equations (7) and (8).

(6) $$\begin{array}{c} \underset{G}{\min}V\left(G\right)=E_{\tilde{x}∼p_{\text{noisy}}\left(\tilde{x}\right)}\left[\log\left(1-D\left(G\left(\tilde{x}\right)\right)\right)\right]+\lambda_{1}L_{\text{dist}}+\lambda_{2}L_{\max}, \end{array}$$

(7) $$\begin{array}{c} L_{\text{dist}}=\sqrt{\overset{N}{\underset{i=1}{\sum }}{\left|{\overset{\wedge }{x}}_{i}-x_{i}\right|}^{2}}, \end{array}$$

(8) $$\begin{array}{c} L_{\max}=\max\left(\left|{\hat{x}}_{1}-x_{1}\right|,\left|{\hat{x}}_{2}-x_{2}\right|,\cdots ,\left|{\hat{x}}_{N}-x_{N}\right|\right), \end{array}$$

where $\tilde{x}\sim p_{\text{noisy}}\left(\tilde{x}\right)$ represents the distribution of the noise signal ($\tilde{x}$), *N* represents the number of samples, ${\hat{x}}_{i}$ represents the denoised signal at sample point *i*, and *x*_*i*_ represents the clean signal at sample point *i*, and *λ*_1_ and *λ*_2_ are the weighting coefficients of *L*_dist_ and *L*_max_, which are experimentally set to 0.7 and 0.2, respectively.

### 2.3. Discriminator and Value Function

**Figure 4:**
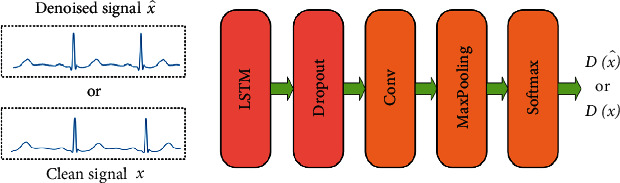
Discriminator structure.

As shown in Figure 4, the discriminator consists of an LSTM layer (size = 128), a dropout layer (rate = 0.5), a convolutional layer (filters = 64, filter size = 16, and stride = 2), a max pooling layer (size = 3 and stride = 2), and a softmax layer.

The optimization goal of the discriminator is to maximize the probability of assigning the correct label to both real examples and generated signals. The value function *V*(*D*) is shown in

(9) $$\begin{array}{c} \underset{D}{\max}V\left(D\right)=E_{x∼p_{\text{data}}\left(x\right)}\left[\log\left(\frac{1}{1+D\left(x\right)}\right)\right]+E_{\tilde{x}∼p_{\text{noisy}}\left(\tilde{x}\right)}\left[\log\left(1-\frac{1}{1+D\left(G\left(\tilde{x}\right)\right)}\right)\right]. \end{array}$$

### 2.4. Experimental Setup

#### 2.4.1. Performance Evaluation Criteria

The improvement of SNR (SNR_imp_), the root mean square error (RMSE), and the percentage root mean square difference (PRD) are used to evaluate the effectiveness of the denoising model. Let *x*_*i*_ denotes the clean ECG signal, ${\tilde{x}}_{i}$ denotes the noisy ECG signal, and ${\hat{x}}_{i}$ denotes the denoised ECG signal. *N* is the length of the proposed signal in one computing cycle.

SNR_imp_ characterizes the SNR difference between the denoised signal and the original clean signal, which is defined as follows:

(10) $$\begin{array}{c} {\text{SNR}}_{\text{imp}}={\text{SNR}}_{\text{out}}-{\text{SNR}}_{\text{in}}, \end{array}$$

where SNR_in_ and SNR_out_ are, respectively, calculated by Equation (11) and Equation (12). From the definition of SNR_imp_, a higher value of SNR_imp_ corresponds to a better performance.

(11) $$\begin{array}{c} {\text{SNR}}_{\text{in}}=10\times \mathrm{lg}\left(\frac{\sum_{n=1}^{N}{x_{i}}^{2}}{\sum_{n=1}^{N}{\left({\tilde{x}}_{i}-x_{i}\right)}^{2}}\right), \end{array}$$

(12) $$\begin{array}{c} {\text{SNR}}_{\text{out}}=10\times \mathrm{lg}\left(\frac{\sum_{n=1}^{N}{x_{i}}^{2}}{\sum_{n=1}^{N}{\left({\overset{\wedge }{x}}_{i}-x_{i}\right)}^{2}}\right). \end{array}$$

RMSE denoted in Equation (13) is used to measure the similarity between the denoised signal and the original clean signal. A smaller value of RMSE corresponds to a higher similarity between the denoised signal and the clean signal.

(13) $$\begin{array}{c} \text{RMSE}=\sqrt{\frac{1}{N}\times \overset{N}{\underset{n=1}{\sum }}{\left(x_{i}-{\overset{\wedge }{x}}_{i}\right)}^{2}}. \end{array}$$

PRD, denoted in Equation (14), is used to measure the error between the reconstructed signal and the target signal. A smaller value of PRD corresponds to a smaller reconstruction loss of the denoising method.

(14) $$\begin{array}{c} \text{PRD}=\sqrt{\frac{\sum_{n=1}^{N}{\left(x_{i}-{\overset{\wedge }{x}}_{i}\right)}^{2}}{\sum_{n=1}^{N}{{\overset{\wedge }{x}}_{i}}^{2}}}\times 100. \end{array}$$

#### 2.4.2. Data Set Experimental Samples

The MIT-BIH Arrhythmia Database [24] and the MIT-BIH Noise Stress Test Database [25] are used to obtain the clean ECG signal data and the noise data, respectively. The MIT-BIH Arrhythmia Database is one of the widely acknowledged standard ECG databases in which a total of 48 records, with 2 channels per record, from 47 subjects were gathered continuously over the course of 24 hours. All ECG signals were digitized at 360 Hz. The MIT-BIH Noise Stress Test Database includes typical noise in 12 half-hour of ECG recordings and 3 half-hour of Holter recordings. Noise recordings are collected from healthy volunteers using a standard ECG acquisition instrument, including BW, MA, and EM. We use the data from the MIT-BIH Arrhythmia Database as clean signals and add noise from the MIT-BIH Noise Stress Test Database to create noisy signals.

*(1) Data Set I*. The experiment data are obtained from 15 records numbered 100, 103, 106, 109, 115, 116, 123, 202, 205, 209, 220, 223, 230, 231, and 234 in the MIT-BIH Arrhythmia Database. We use the modified lead II (ML II) data for each record because lead II is widely used in portable wearable devices. All signals are segmented according to the length of 1024 samples per fragment, and a total of 8254 fragments are acquired. Data set I is used for model comparison, noise reduction of different noise types, method comparison, and individual variability experiments.

*(2) Data Set II*. We also use lead V1 data to verify the denoising performance of the model for other leads. Due to the absence of V1 leads in records 100, 103, and 123, experiment data are obtained from records 106, 109, 115, 116, 202, 205, 209, 220, 223, 230, 231, and 234. All signals are segmented into fragments of length 1024 samples and produced 6381 fragments. The studies using new leads are conducted using data set II.

We choose -1 dB, 3 dB, and 7 dB SNR noise to pollute the original clean ECG signal to test the denoising ability of the model at different SNR levels. Before being utilized as input to the model, all signals are normalized and the data amplitude ranges from 0 to 1. We divide the data sets into the train set (95%) and the test set (5%). The following is the normalizing formula:

(15) $$\begin{array}{c} \text{Norm}\left(x_{i}\right)=\frac{x_{i}-x_{\min}}{x_{\max}-x_{\min}}, \end{array}$$

where *x*_min_ and *x*_max_ are the minimum and maximum values of the sample points.

#### 2.4.3. The Computing Platform

The experimental platform is a Windows environment server with hardware: Intel core i9-9900k CPU at 3.6 GHz with 64 GB of RAM and NVIDIA GeForce RTX2080Ti for computational acceleration. The generative adversarial network is built on Python 3.6 and TensorFlow 2.3. Both the generator and discriminator use the Adam optimizer with an initial learning rate of 0.0001. The batch size is 64.

## 3. Results and Discussion

### 3.1. Experimental Results

#### 3.1.1. Model Comparison

The LSTM is used to correlate the temporal sequence of convolutional features to overcome the limitation of weak mutual information in the small receptive field. We compare our method with DCGAN and LSTM-GAN. These three structures are shown in Figure 2. In addition, three activation functions (ReLU, PReLU, and LeakyReLU) are set to confirm the influence of the activation function on the proposed model.

**Figure 5:**
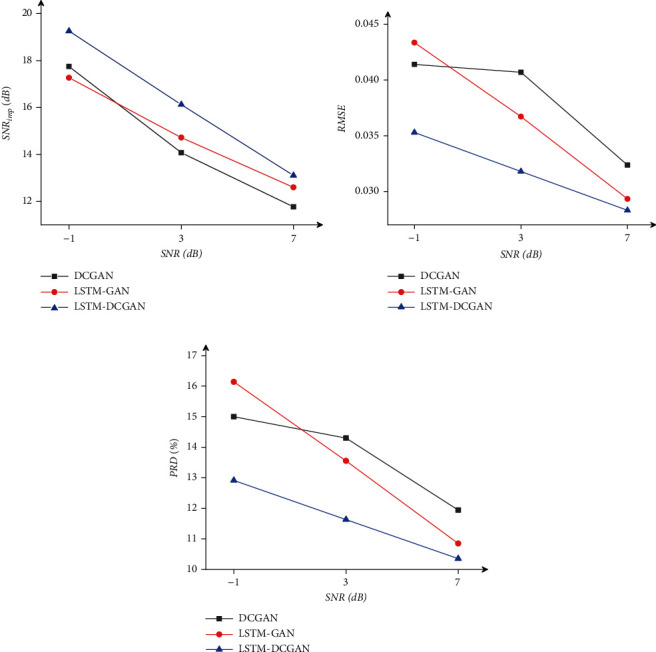
Comparison of denoising results of DCGAN, LSTM-GAN, and LSTM-DCGAN models.

The clean ECG signals for the experiments are from data set I and the noise data from the MIT-BIH Noise Stress Test Database. The noisy ECG signals are formed by adding mixed noise (MA+BW+EM) with different SNR (-1 dB, 3 dB, and 7 dB) to clean ECG signals. The SNR_imp_, RMSE, and PRD indicators are used to evaluate the performance of the model. The results of the model structure comparison are shown in Figure 5. The experimental results of the comparison of the three different activation functions in the proposed model are shown in Table 1.

It can be seen from Figure 5 that our proposed convolution combined with LSTM adversarial method has higher SNR_imp_ and lower RMSE and PRD, regardless of the level of noise pollution. In addition, it can be seen from Table 1 that the model with LeakyReLU as the activation function outperforms the other two models.

#### 3.1.2. Noise Reduction of Different Noise Types

To measure the denoising ability of the model for different noise, we add MA, BW, EM MA+BW, MA+EM, BW+EM, and MA+BW+EM with different SNR levels (-1 dB, 3 dB, and 7 dB) to the clean ECG signals in data set I.

Table 2 shows our method's average SNR_imp_, RMSE, and PRD scores for different kinds of noise removal at -1 dB, 3 dB, and 7 dB. For single noise removal, the SNR_imp_ of our method reaches 19.873 dB, and for mixed noise removal, it reaches 19.254 dB. The results in Table 2 show that our method can remove not only single noise but also multiple mixed noise. Because EM noise can imitate the appearance of ectopic beats, the results of our model for removing EM noise are not as excellent as other noise.

**Figure 6:**
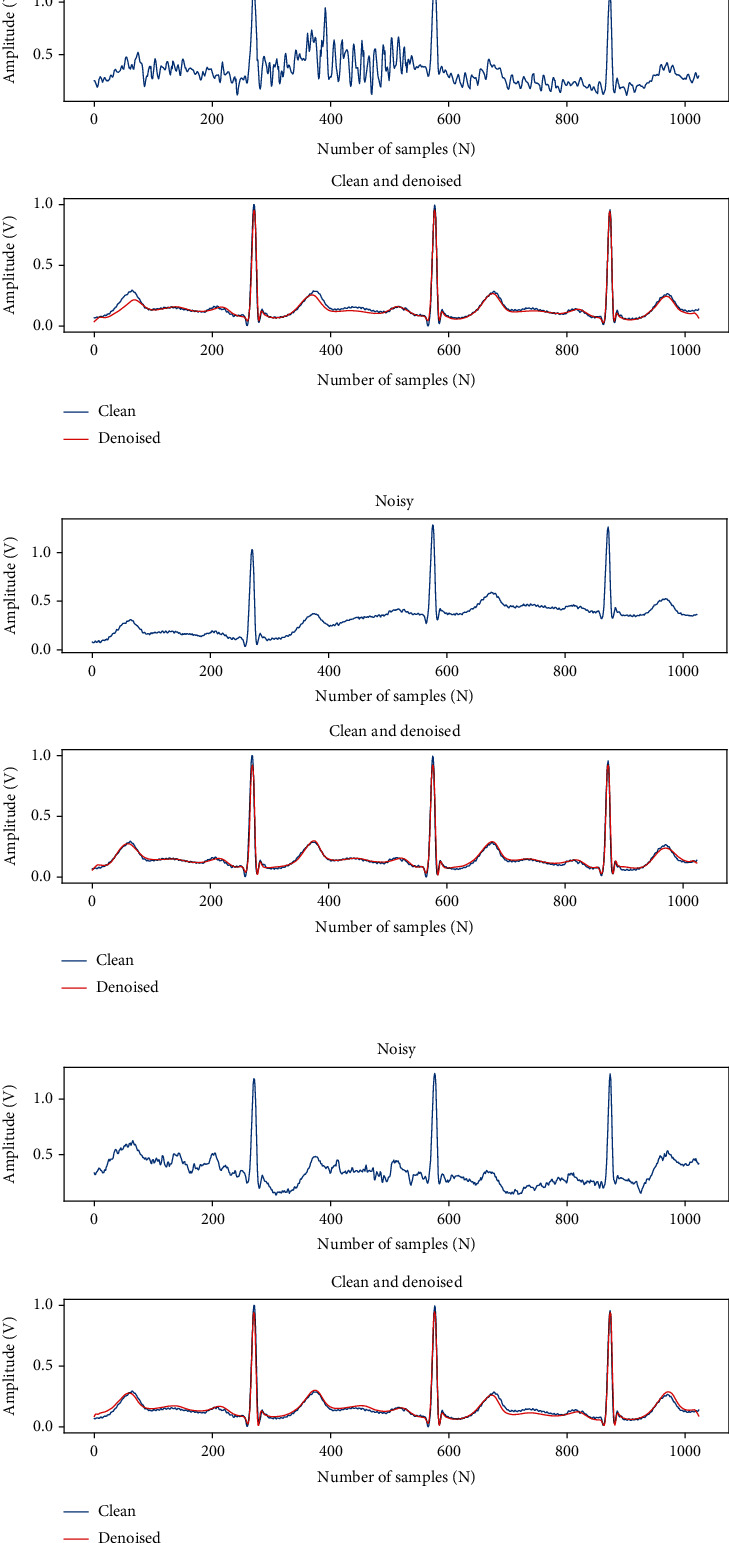
The results of removing the three kinds of single noise.

Figure 6 shows the results of our model for removing EM, BW, and MA. In each subplot, the first row represents the noisy ECG signal. The second row is the comparison between the clean signal (blue) and the denoised signal (red).

**Figure 7:**
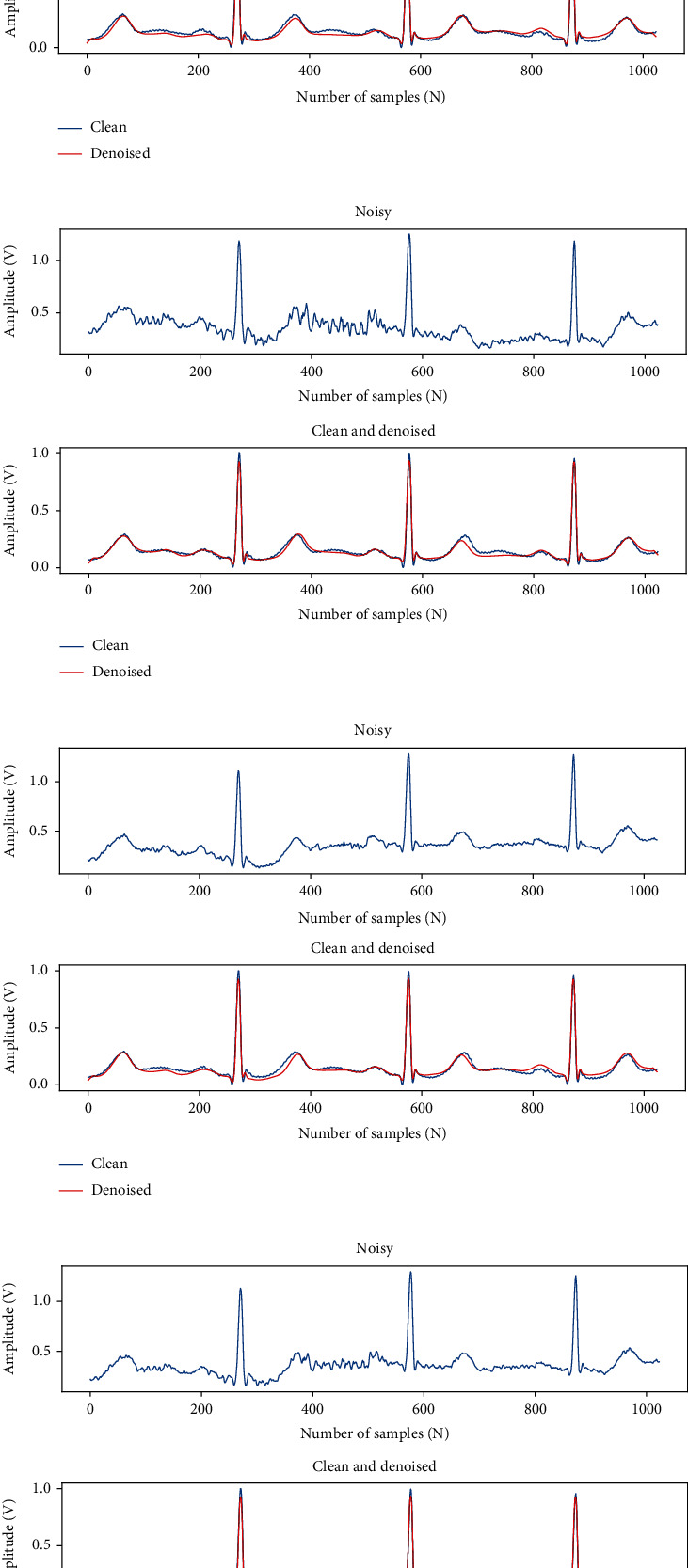
The results of removing the four kinds of mixed noise.

Figures 7(a)–7(d) represent the results of our method for removing four kinds of mixed noise, MA+BW, MA+EM, BW+EM, and MA+BW+EM. In each subplot, the first row represents the ECG signal with mixed noise, and the second row compares the denoised signal (red) and the original clean signal (blue). The overall denoised ECG signal is close to the original clean ECG signal, and the experimental results show that our method can also effectively reduce the various mixed noise contained in the ECG signal.

#### 3.1.3. Method Comparison

We compare the proposed method with the state-of-the-art method GAN and FCN-DAE. In GAN [18], the structure of the generator network has the number of nodes 310-250-250-310, and the number of nodes of the discriminator network structure is 310-150-150-1. In the generator and discriminator, a layer of tanh activation function is added after each hidden layer, and the activation function of the output layer is a sigmoid function. In FCN-DAE [16], it includes an encoder (filters = 40 − 20 − 20 − 20 − 40 − 1) composed of convolutional layers and a decoder (filters = 1 − 40 − 20 − 20 − 20 − 40 − 1) composed of deconvolution layers, and the convolutional kernel size of both encoder and decoder is 16. All methods used clean ECG signal from data set I, and the test set and train set remain consistent. Because the daily collected ECG signal often contains a variety of noise, noisy ECG signals are formed by adding mixed noise (MA+BW+EM). SNR_imp_, RMSE, and PRD are used to measure the performance of all methods.

**Figure 8:**
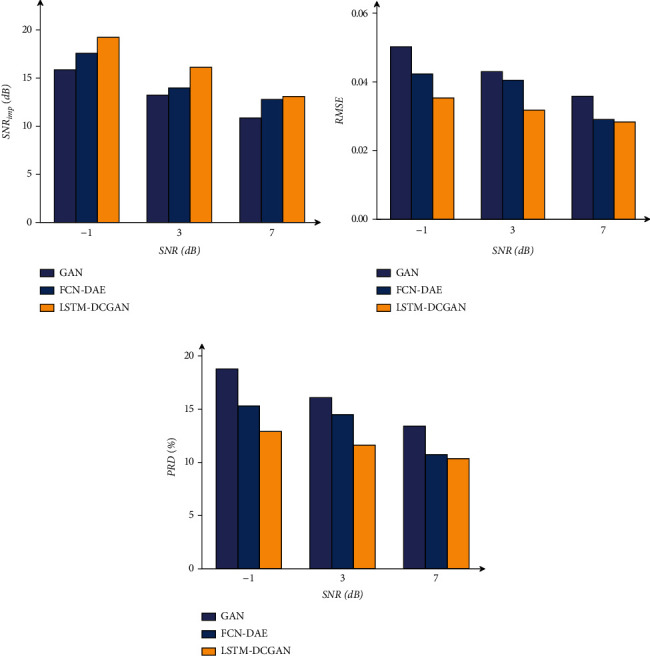
Comparison of denoising performance of all evaluated methods at different input SNR levels.

Figures 8(a)–8(c) show the average SNR_imp_, RMSE, and PRD scores of the three denoising models. The SNR characterizes the noise level of the signal; a higher value of the SNR corresponds to less noise in the signal. As can be seen from Figure 8(a), for each denoising method, the SNR_imp_ decreases as the SNR increases. This is consistent with our expectation that as the noise decreases, the degree of noise signal removal also decreases. The RMSE and PRD represent the closeness between the denoised signal and the original clean signal. The lower values of RMSE and PRD correspond to less distortion of the denoised signal. When the SNR is -1 dB, the SNR_imp_ scores of the three methods are 15.870 dB, 17.569 dB, and 19.254 dB; the RMSE scores are 0.050, 0.043, and 0.035; the PRD scores are 18.77, 15.32, and 12.91. Regardless of the level of SNR, the SNR_imp_ of our method is higher than that of GAN and FCN-DAE, and the RMSE and PRD of our method are lower than the other two methods, which means the denoising performance of our method is better than the other two methods.

**Figure 9:**
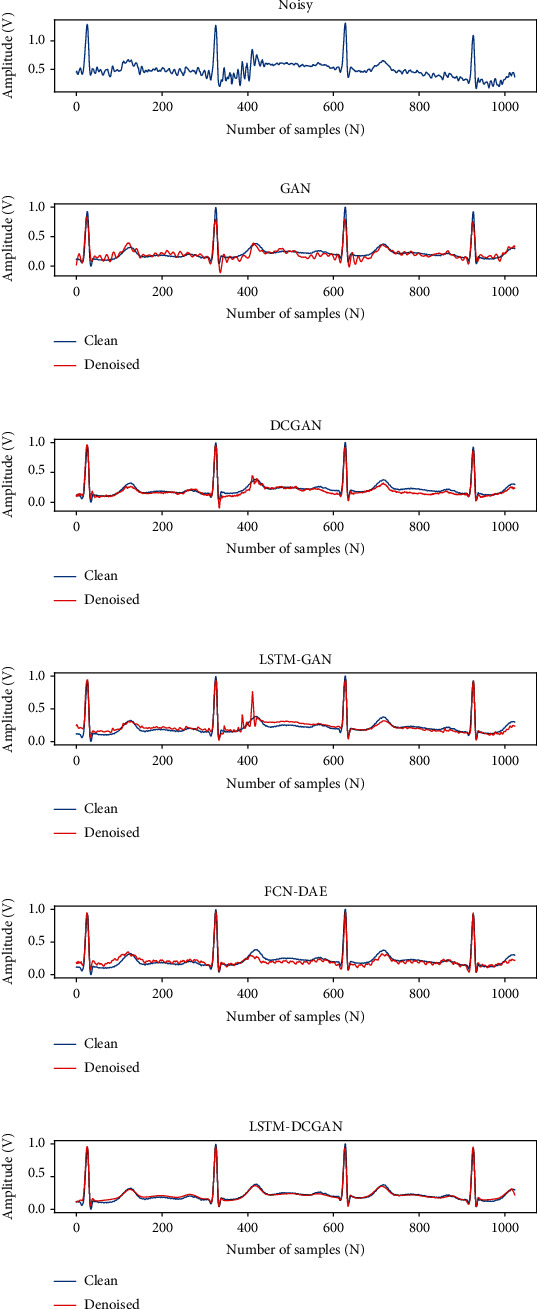
The denoising results of GAN, DCGAN, LSTM-GAN, FCN-DAE, and LSTM-DCGAN.

In Section 3.1.1 and Section 3.1.3, we compare the SNR, RMSE, and PRD indicators of the five methods, and the indicators show that our method outperforms several other methods. In Figure 9, we plot the denoising waveforms of the five methods to observe the denoising of the signal. From Figure 9, it can be seen that GAN has a significant cut on the R-wave, which makes the amplitude of the R-wave significantly lower than that of the original clean signal, and the overall waveform has small spurs. The DCGAN can effectively remove the noise from the signal, and the overall waveform is smooth. The R-wave also has a high degree of coincidence with the clean signal but does not match the original waveform in some details. Although LSTM-GAN can reduce very small noise, it is not effective in reducing large amplitude noise. Using FCN-DAE for denoising, the coincidence with the original clean signal is better than the above three methods, but the waveform is not smooth enough. Among the five methods, our method possesses a higher coincidence with the original clean signal while removing the noise. The above results show that our method also outperforms the other methods in denoising waveforms.

### 3.2. Discussion

#### 3.2.1. Discussion of the Results of the Model

**Figure 10:**
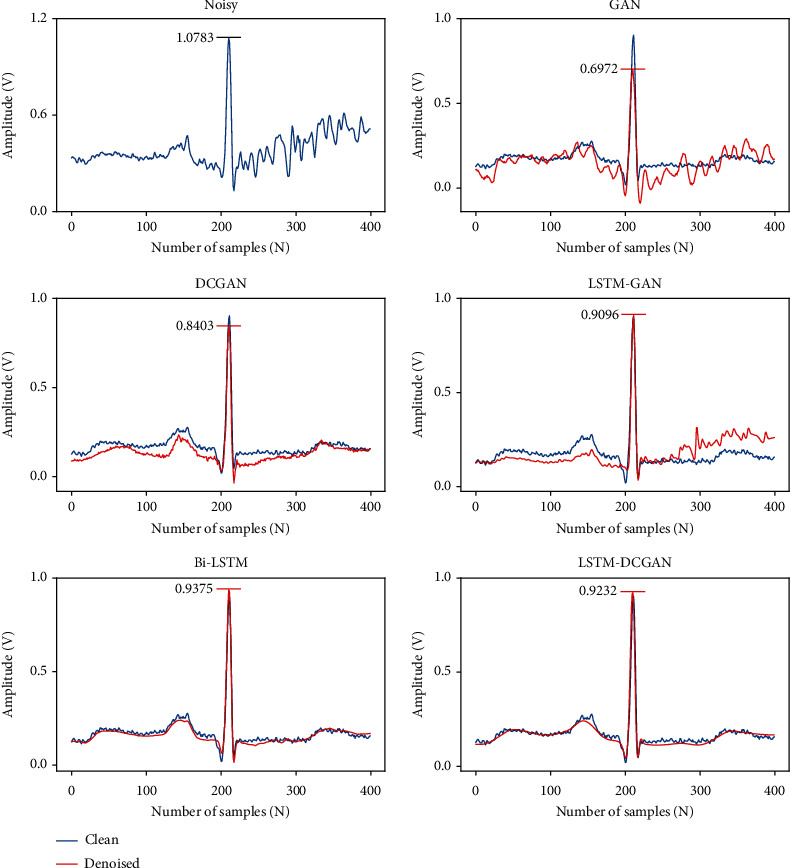
Comparison of denoising detailed waveforms GAN, DCGAN, LSTM-GAN, Bi-LSTM, and LSTM-DCGAN.

In this section, we analyze the ECG denoising results for generative adversarial networks with different structures and Bi-LSTM networks.  Figure 10 shows the denoised waveforms of one complete ECG cycle for the five networks. We can observe that GAN has the obvious cutting of R-waves in ECG waveforms, the denoised waveforms of DCGAN have low coincidence with the original clean signal, and LSTM-GAN is not effective enough for high-level noise reduction. Our method can not only remove some slight noise but also remove high-level noise. In addition, the denoised signal obtained by our method is smoother and has a higher coincidence with the original clean signal. We analyze the reasons for this result. Feature extraction is performed by the local convolutional kernel. Each convolution operation is performed on the receptive field, and each neuron only considers the information in the receptive field. A smaller receptive field corresponds to a smaller kernel size and stride, and more detailed features can be extracted. For the whole denoising process, deeper detailed information can be preserved. This is why many researchers choose smaller kernel sizes and stride sizes when designing convolutional neural networks, but convolutional kernels with smaller receptive fields are less capable of grasping global information. Therefore, the convolution-based signal denoising method is the reconstruction from local detailed information to global information. To consider global information, we start from two points: firstly, the adversarial strategy is employed. The adversarial approach begins with the overall data distribution to give the denoised signal access to the same distribution as the clean signal. Secondly, the global information is poorly preserved for the convolution of small size filters. The LSTM layer is added after each convolutional layer in our generative adversarial structure to associate the order dependence of the convolved features.

It can be seen from Figure 10 that the Bi-LSTM model can effectively remove the noise in the signal. To further verify the advantages and disadvantages of the two methods, we compare the denoising results of Bi-LSTM and LSTM-DCGAN for different kinds of noise (MA, BW, and EM) at various SNR. From the results in Table 3, Bi-LSTM outperforms our method in removing MA noise but performs worse in removing BW and EM noise. The SNR_imp_ of Bi-LSTM for the three types of noise removal ranges from 15.063 dB to 20.807 dB, and the difference in the removal of different noise reaches 5.744 dB, while the difference of our method is only 1.932 dB.

#### 3.2.2. Robustness of Model Deployment

**Figure 11:**
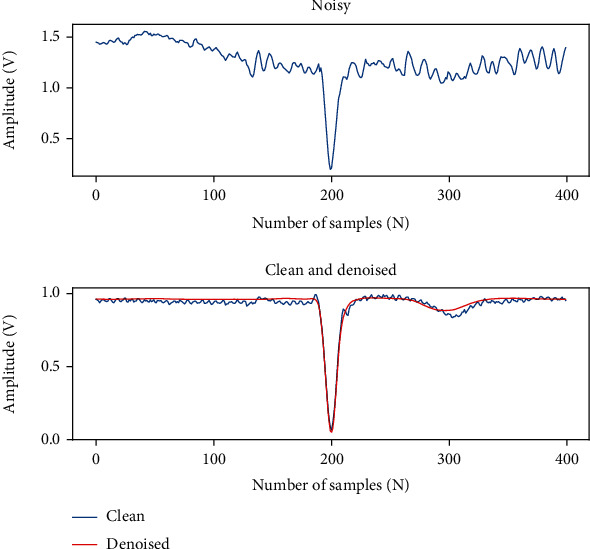
Denoising waveform of lead V1.

We choose the ECG signal in data set II to confirm the applicability of our proposed methods to other leads. MA+BW+EM mixed noise was added to the original V1 original signal at -1 dB, 3 dB, and 7 dB.  Table 4 shows the results of the noise removal for the new leads.  Figure 11 shows the waveform plot of the model's denoising results for lead V1.

From the results in Table 4, the denoising results of V1 leads are slightly worse than the lead II leads at -1 dB and 7 dB and slightly better than the lead II at 3 dB. The differences in SNR_imp_ between the two leads at -1 dB, 3 dB, and 7 dB are 3.33%, 4.48%, and 9.67%, respectively. As a result, our method can be used to remove noise from ECG signals in other leads within an acceptable range.

The effectiveness of the model in denoising ECG signal differences in various individuals was tested. A 15-fold repeated experiment is designed with experimental data from the MIT-BIH Arrhythmia Database of 15 different patients; they are recorded as 100, 103, 106, 109, 115, 116, 123, 202, 205, 209, 220, 223, 230, 231, and 234. Of the 15 sets of records, 14 of them are used for model training, and the remaining 1 is used for model testing, thus repeating the experiment 15 times to observe the model's ability to process “new data.” Since the ECG signals collected daily are often accompanied by various noise disturbances, the clean signals are polluted with a mixture of MA+EM+BW noise to form noisy ECG data.

The results in Table 5 show that the denoising effect of each lead varies due to individual differences, with the SNR_imp_ of the patient data of 115 records reaching 19.217 dB and the result of the patient data of 202 records being lower, only 11.900 dB. This is mainly caused by the data dependence of the model, which is unable to learn information about data that is not involved in training. To reduce the data dependency of the model, we use a small amount of new data for model boosting to obtain information about the new data. Model boosting is performed by adding a small number of samples to the existing model. Take the worst record 202 as an example, by adding 20% of the data (108 samples) into the model training and 80% (434 samples) as the test of the boosted model. The SNR_imp_ for lead 202 is increased from 11.900 dB to 15.237 dB.

## 4. Conclusions

We propose a GAN method using CNN combined with LSTM to draw attention to local and global information in ECG denoising studies. Convolution is used throughout the network structure to help the denoised signal preserve more detailed information. In order to make the global information highly preserved, we employ the generative adversarial strategy to guarantee that the data distributions of the original clean signal and the denoised signal are consistent. Additionally, we update the generator and discriminator of the GAN by adding an LSTM layer after each convolutional layer to provide time series information to the features. According to experimental results, our technique has a higher SNR_imp_, a smaller RMSE, and a lower PRD than DCGAN and LSTM-GAN. The comparison of denoised waveforms also demonstrates that our method outperforms the other two models in terms of noise suppression and detailed preservation. However, our method requires a large amount of computation. Another important area for research in the future is lightweight denoising models, which are being used for portable wearable devices. In addition, there are many additional active and effective ECG-denoising models that need to be validated.

## Figures and Tables

**Table 1:** Effect of three different activation functions on the proposed model.

Indicators	Activation function		
	ReLU	PReLU	LeakyReLU
SNR_imp_	17.043	18.263	19.254
RMSE	0.045	0.040	0.035
PRD	16.440	14.381	12.914

**Table 2:** The average SNR_imp_, RMSE, and PRD of denoising results with different kinds of noise.

SNR	Indicators	Noise type						
		MA	BW	EM	MA+BW	MA+EM	BW+EM	MA+EM+BW
-1 dB	SNR_imp_	18.334	19.873	17.941	18.403	18.429	19.157	19.254
	RMSE	0.039	0.033	0.041	0.040	0.039	0.036	0.035
	PRD	14.140	12.117	15.140	13.981	14.074	13.086	12.914

3 dB	SNR_imp_	16.083	16.249	14.935	16.094	15.323	15.856	16.125
	RMSE	0.032	0.032	0.036	0.032	0.035	0.033	0.032
	PRD	11.762	11.516	13.439	11.817	12.661	12.033	11.629

7 dB	SNR_imp_	13.036	13.119	12.154	12.332	12.823	12.534	13.105
	RMSE	0.028	0.028	0.032	0.031	0.029	0.0301	0.028
	PRD	10.469	10.584	11.658	11.148	10.677	11.180	10.350

**Table 3:** The results of removing different kinds of noise by Bi-LSTM and LSTM-DCGAN.

Indicators	Methods	Noise type		
		MA	BW	EM
SNR_imp_	Bi-LSTM	20.807	18.896	15.063
	LSTM-DCAGN	18.334	19.873	17.941

RMSE	Bi-LSTM	0.029	0.036	0.057
	LSTM-DCAGN	0.039	0.033	0.041

PRD	Bi-LSTM	10.904	13.525	20.724
	LSTM-DCAGN	14.140	12.117	15.140

**Table 4:** Denoising results of the model for lead V1.

Indicators	Lead	SNR		
		-1 dB	3 dB	7 dB
SNR_imp_	V1	18.632	16.827	11.950
	MLII	19.254	16.125	13.105

RMSE	V1	0.045	0.029	0.033
	MLII	0.035	0.032	0.028

PRD	V1	14.276	11.148	11.808
	MLII	12.914	11.629	10.350

**Table 5:** Denoising ability of 15-fold experiments on new data.

Indicators	Records															
	100	103	106	109	115	116	123	202	205	209	220	223	230	231	234	Average
SNR_imp_	14.473	16.291	14.380	13.728	19.217	16.587	19.163	11.900	14.549	17.745	17.995	15.419	18.443	13.389	14.355	15.842
RMSE	0.044	0.036	0.052	0.082	0.041	0.048	0.042	0.055	0.042	0.056	0.053	0.045	0.058	0.074	0.043	0.051
PRD	21.363	17.452	21.991	23.210	12.611	16.953	12.597	29.069	21.212	14.909	14.266	19.198	13.650	24.258	21.830	18.971

## Data Availability

All the data utilized in our research can be accessed from https://physionet.org/content/mitdb/1.0.0/.
